# Amelioratory Effect of Resistant Starch on Non-alcoholic Fatty Liver Disease *via* the Gut-Liver Axis

**DOI:** 10.3389/fnut.2022.861854

**Published:** 2022-05-17

**Authors:** Weifeng Zhu, Ying Zhou, Rong Tsao, Huanhuan Dong, Hua Zhang

**Affiliations:** ^1^Department of Food Nutrition and Safety, College of Pharmacy, Jiangxi University of Traditional Chinese Medicine, Nanchang, China; ^2^Guelph Research and Development Centre, Agriculture and Agri-Food Canada, Guelph, ON, Canada

**Keywords:** resistant starch, NAFLD, gut-liver axis, gut microbiota, gut metabolites

## Abstract

Non-alcoholic fatty liver disease (NAFLD) is a hepatic manifestation of metabolic syndrome with a global prevalence. Impaired gut barrier function caused by an unhealthy diet plays a key role in disrupting the immune-metabolic homeostasis of the gut-liver axis (GLA), leading to NAFLD. Therefore, dietary interventions have been studied as feasible alternative therapeutic approaches to ameliorate NAFLD. Resistant starches (RSs) are prebiotics that reduce systemic inflammation in patients with metabolic syndrome. The present review aimed to elucidate the mechanisms of the GLA in alleviating NAFLD and provide insights into how dietary RSs counteract diet-induced inflammation in the GLA. Emerging evidence suggests that RS intake alters gut microbiota structure, enhances mucosal immune tolerance, and promotes the production of microbial metabolites such as short-chain fatty acids (SCFAs) and secondary bile acids. These metabolites directly stimulate the growth of intestinal epithelial cells and elicit GPR41/GPR43, FXR, and TGR5 signaling cascades to sustain immune-metabolic homeostasis in the GLA. The literature also revealed the dietary-immune-metabolic interplay by which RSs exert their regulatory effect on the immune-metabolic crosstalk of the GLA and the related molecular basis, suggesting that dietary intervention with RSs may be a promising alternative therapeutic strategy against diet-induced dysfunction of the GLA and, ultimately, the risk of developing NAFLD.

## Introduction

Diets high in sugar and fats cause microbiota dysbiosis, which impairs gut immune tolerance and contributes to increased risk of metabolic disorders (1). Being the primary metabolic organ, high-fat diet (HFD)-induced impairment in the metabolic profile of the liver can promote lipogenesis and inhibit free fatty acid (FFA) oxidation, which eventually progresses to non-alcoholic fatty liver disease (NAFLD) (2). NAFLD is a hepatic manifestation of metabolic syndrome and has become one of the most common causes of liver disease worldwide (3), accounting for a considerable burden on healthcare systems (4). Intracellular fat accumulation-induced steatosis and altered metabolic homeostasis are the primary features of NAFLD (5). A high prevalence of NAFLD (33.6%) was observed in patients with inflammatory bowel disease (IBD) (6). This suggests that an integrated coordination of the gut-liver axis (GLA) exists and is important for the maintenance of immune-metabolic homeostasis. This makes the GLA a promising therapeutic target for treating NAFLD.

As disturbances in gut integrity and dysbiosis impair the physiological function of the liver along the GLA, restoration of the microenvironment in the lower gut can be a potential and efficacious approach to ameliorating NAFLD (7), including dietary interventions aimed at maintaining gut microbiota composition, mucosal function, and barrier integrity, particularly prebiotics. Resistant starches (RSs) have been widely found in food sources rich in carbohydrates, such as corn, potato, and banana, which are often processed into a broad variety of foods (i.e., breads, cereals, pasta, snacks, and beverages). RSs are indigestible carbohydrates but fermentable for gut microbiota; thus, they are widely believed to be effective prebiotics that improve the production of short-chain fatty acids (SCFAs), which benefits the gut microbiome structure and overall human health (8). Current findings suggest that dietary supplementation with probiotics, functional oligosaccharides, and dietary fibers can help maintain gut bacterial balance and improve immune homeostasis in the gut, which is potentially beneficial for NAFLD amelioration (5, 9). However, the role and underlying mechanism of RSs in ameliorating NAFLD by enhancing gut microenvironment homeostasis remain largely unknown. This review aims to provide insights into RSs as a dietary strategy to alleviate liver disease conditions of NAFLD, with a particular focus on intestinal microecological changes from the perspective of the GLA (10).

## Pathogenesis of Non-Alcoholic Fatty Liver Disease Via the Gut-Liver Axis

**FIGURE 1:**
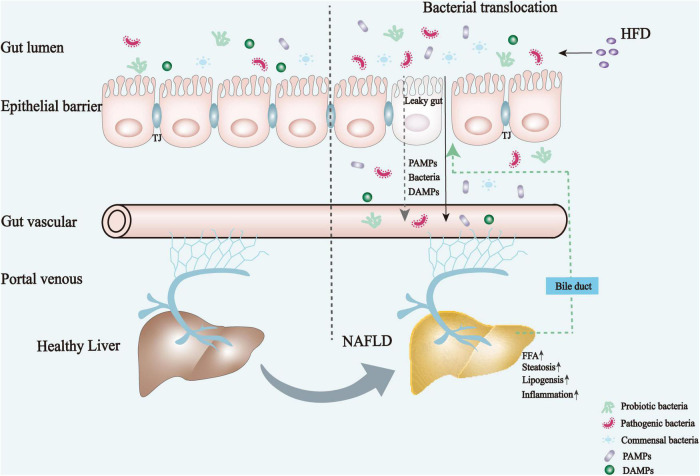
Mechanism underlying the pathogenesis and physiological alterations of NAFLD. High-caloric diets lead to an imbalanced intestinal flora, which in turn elicits an impaired gut barrier function and increased permeability, followed by bacterial translocation and an increase of harmful metabolites or bacterial products that eventually enter the liver through the portal vein. These factors together promote the development of NAFLD by exacerbating hepatic steatosis, lipogenesis, and inflammatory responses. DAMPs, damage-associated molecular patterns; FFA, free fatty acid; HFD, high-fat diet; TJ, tight junctions; PAMPs, pathogen-associated molecular patterns.

The underlying mechanisms for the development and progression of NAFLD are complex. The interdependence between the gut and liver forms a close integration of their molecular and physiological functions, playing a key role in the integrated pathogenesis of NAFLD, as shown in Figure 1 (10, 11). The excessive intake of high calories promotes the accumulation of fat in visceral and subcutaneous adipose tissues, where the amounts of FFAs and total glycerol are dramatically increased. As such, NAFLD eventually developed. Moreover, there is a clear causal link between NAFLD and dysbiosis of the gut microbiota. Patients with NAFLD tend to have increased intestinal permeability and microbiota dysbiosis (12, 13). Dysfunction and dysregulation of the intestinal barrier and dysbiosis impair mucosal immune tolerance, leading to systemic inflammation and disturbing liver immune metabolism homeostasis.

The intestinal barrier is a complex functional unit composed of lumen and mucosal components (i.e., epithelial cell layer, mucosal barrier, and innate and acquired immune components), neurointestinal, vascular, and endocrine systems, digestive enzymes, and gut microbiota (14). In addition to the epithelial layer and mucus, recent evidence has characterized the gut-vascular barrier, which prevents the translocation of bacteria directly into portal circulation (15). However, the loss of gut barrier integrity and mediated translocation of the gut microbiome evokes a toll-like receptors (TLR)-mediated pro-inflammatory cascade in the liver (16–18). In addition, pathogenic bacteria from the intestinal microbiota can interactively regulate IL-17A production from immune or non-immune cells, which plays a major role in regulating gut mucosal immunity and pathogenesis of NAFLD, and thus accelerates the progression of NAFLD, a highly related complication of atherosclerosis (19–22). Moreover, pathogenic bacterial metabolites [i.e., lipopolysaccharides (LPS) and ethanol] from the lumen to the circulation rapidly relay information to the brain and damage the periphery, mainly in the liver and adipose tissues, by altering the central neurotransmitter systems (5). The vagus nerve in the gut can be directly activated by inflammatory signals to impair insulin sensitivity and hepatic steatosis associated with liver inflammation by altering the central neurotransmitter system (23). Available research demonstrates the role of enterohepatic axis dysfunction in the development of NAFLD; the underlying mechanisms can be summarized as: (1) alterations in the gut microbiome profile and immune responses; (2) the effects of gut bacterial components and metabolites, such as LPS, endogenous ethanol (EnEth), and SCFAs; and (3) the impairment of intestinal barrier function and bile acid (BA) homeostasis (24). Owing to the inflammatory tone, metabolic homeostasis and functionality of the liver are impaired, leading to an increased risk of developing metabolic disorders, particularly NAFLD.

## Resistant Starch

**FIGURE 2:**
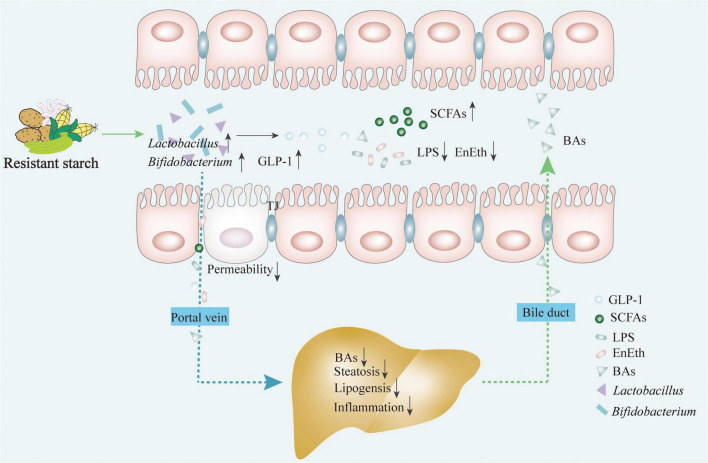
RSs exert the effects on ameliorating NAFLD via restoring the gut microbiota structure and regulating bacterial metabolites through the link between gut and liver. Intake of RSs contributes to: (1) improving the growth of probiotics (e.g., *Lactobacillus* and *Bifidobacterium*); (2) promoting the production of metabolites (e.g., short chain fatty acids and glucagon-like peptide-1); (3) inhibiting harmful metabolites production (e.g., LPS and EnEth); and (4) maintaining the homeostasis of the BAs. This regulates the enterohepatic axis homeostasis by modulating flora metabolite and intestinal hormone productions to inhibit hepatic steatosis, lipogenesis, and inflammatory responses. BAs, bile acids; EnEth, endogenous ethanol; GLP-1, glucagon-like peptide-1; LPS, lipopolysaccharide; RS, resistant starch; SCFAs, short-chain fatty acids.

Resistant starches are defined as the total amount of starch and starch degradation products that resist digestion in the small intestine, and are therefore recognized as a typical prebiotic (25). Naturally occurring RSs are widely found in cereal grains, seeds, heated starches, and starch-containing foods (26). Furthermore, RSs are classified into five types (RS1–RS5) according to their source and processing procedure. RS1 are starch granules that occur in some indigestible plant materials, such as whole grains; RS2 are native granular starches, such as raw potatoes, green bananas, gingko, or high-amylose maize; RS3 are retrograded amylose starch or crystallized starches, such as cooked and cooled starchy foods; RS4 are chemically modified starches produced via esterification, cross-linking, or transglycosylation; and RS5 are amylose-lipid complex, amylose, and long branch chains of amylopectin from single-helical complexes with fatty acids and fatty alcohols when the starch molecules interact with lipids (27, 28). As humans do not have the enzymes to digest RSs, gut microbes ferment RSs to benefit the host by selectively stimulating the growth of intestinal epithelial cells and probiotic strains in the lower gut, thereby improving the overall health of the host (29). The gut bacterial fermentation of prebiotics increases the concentration of SCFAs in the cecum and portal vein blood, which are eventually transported through the blood circulation to various internal organs and tissues, as shown in Figure 2. Simultaneously, the altered intestinal metabolomic profiles and associated bioactive metabolites may be involved in the regulation of signaling cascades in the GLA, exerting beneficial effects on the host (Figure 2). Emerging evidence from animal studies strongly demonstrates the efficacy of RSs in the prevention or treatment of various diseases [e.g., IBD, inflammatory bowel syndrome, colon cancer, obesity, type 2 diabetes mellitus (T2DM) and cardiovascular disease]; however, the data in humans remain ambiguous. The possible mechanism of RSs in ameliorating NAFLD from the perspective of the GLA is still unknown, warranting further in-depth studies.

## Therapeutic Potential of Resistant Starches on Ameliorating Non-Alcoholic Fatty Liver Disease

Lifestyle interventions, such as eating a healthy diet and regular exercise, are among the most effective and safe ways to mitigate NAFLD, as well as other types of metabolic disorders. Recent evidence has highlighted the preventive and therapeutic effects of some plant foods, particularly those rich in bioactive polyphenols, carotenoids, oleic acid, n-3 polyunsaturated fatty acids, and fiber (30–32). RSs, being food components, have physiological properties similar to those of fermentable dietary fibers. It has been found that RS intake reduces fat accumulation to improve insulin sensitivity, thereby maintaining blood glucose levels and lipid metabolic homeostasis (27, 33). A human study confirmed that RSs significantly improved insulin and low-density lipoprotein cholesterol (LDL-C) levels in obese patients (34). Furthermore, supplementation of green bananas rich in RSs in NAFLD model mice was shown to improve SCFAs production and reduce hepatic steatosis by regulating the transporters involved in lipid excretion and adipogenesis (35). A previous finding illustrated that RSs exhibit the ability to lower serum cholesterol by interacting with BAs, which might be related to the increased expression of hepatic cholesterol 7α-hydroxylase (CYP7A1) and fecal BA excretion (33). Overall, RSs may be a promising dietary approach for the alleviation of NAFLD by maintaining lipid metabolic homeostasis (Table 1). However, the understanding of how RS intake contributes to ameliorating NAFLD remains scarce. It is necessary to explore the molecular basis of RSs sustaining the integrated gut homeostasis involved in the symbiotic microbiota, mucosal immune response, and metabolism toward prevention or mitigate NAFLD. Moreover, clinical studies are needed to investigate the regulatory effects of RS intake in patients with NAFLD.

**TABLE 1:** Effect of RS on NAFLD.

	Model	Dose	Time	NAFLD-related parameters	References
Rice starch-oleic acid complex	Male Sprague-Dawley rats (non-obese) fed HFD	54.5% in HFD	8 weeks	TG↓; TC↓; HDL-C↑; SOD↑; GSH-PX↑; MDA↓; AST↓; and ALT↓	(47)
Raw potato starch	Duroc × Landrace × Large White growing barrows	230 (growing) or 280 g/kg (finishing)	100 days	Fatty acid biosynthesis↓; acid β-oxidation↑; FABP1↑; fatty acid intake ↓; fatty acid synthesis↓; fatty acid oxidation↑; and glycerophospholipid synthesis↑	(97)
High amylose maize starch	Male Wistar rats (non-obese) fed a high-fat, high-sucrose diet	2 g/day	6 weeks	Blood glucose↓; TC↓; TG↓; HDL-C↑; LDL-C↓; T-AOC↑; T-SOD↑; MDA↓; GSH-PX↑; AST↓; ALT ↓; AKP↓; SREBP-1↓; FAS↓; LXRα↓; and FABP4↓	(98)
Purple yam RS	Male golden hamsters fed HFD	0.5 g/100 g, 1.5 g/100 g	4 weeks	HDL-C↑; TG↓; TC↓; LDL-C↓; and liver fat accumulation↓	(99)
Maize RS	Male Sprague–Dawley rats fed HFD	1.2 g/day	6 weeks	Liver weight ↓; TG↓; TC↓; LDL-C↓; PPAR-γ↓; LXR↓; SREBP-c↓; FAS↓; and ACC↓	(100)
Maize RS	Female ob/ob mice fed HFD	10%, 15%, and 20% in HFD	12 weeks	Liver weight↓; lipid droplet accumulation↓; TBA↓; LPS↓; TG↓; TC↓; AST↓; ALT↓; PPAR↑; and AMPK pathways↑	(86)
Microwave–toughening treatment starch	Male C57BL/6J (B6) mice fed HFD	MTT starch in HFD	5 weeks	Liver index↓; fasting glucose↓; and fat vacuoles↓	(101)
Sorghum RS	Female Sprague–Dawley rats given Formestane for 50 mg/kg BW/d, fed with no soy feed or ordinary feed	0.9 g/kg, 1.8 g/kg, and 2.7 g/kg	6 weeks	Liver steatosis↓; FXR↓; SREBP-1↓; ACC↓; FAS↓; and SCD1↓;	(102)
Green banana (*Musa* sp.) RS	Male C57BL/6 mice fed HFD	15% RS in HFD	14 weeks	Liver steatosis↓; fasting glucose↓; HOMA-IR↓; TG↓; TC↓; p-AMPK/AMPK↑; and HMG CoA-R↓	(35)
Buckwheat RS	Male C57BL/6 mice fed HFD	33.4% RS in HFD	6 weeks	Liver index ↓; HDL-C↑; TG↓; TC↓; LDL-C↓; IL-6↓; TNF-α↓; LPS↓; SOD↑; T-AOC↑; and MDA↓	(65)
Maize RS	Male Wistar rats fed HFD	41.6% RS in HFD	9 weeks	TG↓;TC↓;NEFA↓; HOMA-IR; IRS1↓; IRS2↓; and PPARGC1α↑	(103)
High amylose starch or Esterified high amylose starch	Male Wistar rats (non-obese) fed a high-fat	2 g/day	6 weeks	TC↓; TG↓; HDL-C↑; LDL-C↓; AST↓; ALT↓; MDA↓; GSH-PX↑; T-AOC↑; T-SOD↑; ACC↓; SREBP-1↓; PPAR γ↓; and LXRα↓	(104)

*ACC, acetyl CoA carboxylase; ALT, alanine aminotransferase; AMPK, adenosine 5′-monophosphate (AMP)-activated protein kinase; AST, aspartate aminotransferase; FABP, fatty acid-binding protein; FAS, fatty acid synthase; GSH-Px, glutathione peroxidase; HDL-C, high density lipoprotein cholesterol; HFD, high-fat diet; HMG-CoA, 3-hydroxy-3-methylglutaryl coenzyme A; HOMA-IR, homeostatic model assessment for insulin resistance; IL-6, interleukin-6; ISR, insulin receptor substrate; LDL-C, low density lipoprotein cholesterol; LPS, lipopolysaccharides; LXR, liver X receptors; MDA malondialdehyde; NEFA, non-esterified fatty acid; PPAR, peroxisome proliferators-activated receptors; PPARGC1α, peroxisome proliferator-activated receptor, gamma, coactivator 1 alpha; RS, resistant starch; SCD1, stearoyl-CoA desaturase-1; SOD, superoxide dismutase; SREBP, sterol regulatory element-binding protein; T-AOC, total antioxidant capacity; TBA, total bile acids; TC, total cholesterol; TG, triglyceride; TNF-α, tumor necrosis factor-α; T-SOD, total superoxide dismutase.*

*The symbol ↑ is upregulated, and the symbol ↓ is downregulated.*

## Potential Mechanisms of Resistant Starches on Regulating the Gut-Liver Axis Homeostasis Toward Non-Alcoholic Fatty Liver Disease Mitigation

The intestinal microecology, consisting of intestinal microbiota, intestinal epithelial cells, and the immune system, may play a role in energy metabolism (36). Recent human and rodent studies on obesity-related metabolic disorders have suggested that the gut microbiome plays a key role in NAFLD pathogenesis (37). The long-term consumption of diets high in calories and saturated fat may lead to dysbiosis in the gut microbiota. This, in turn, would evoke an imbalance in the BA pool and a dysfunctional intestinal barrier, followed by increased translocation of bacteria and accumulation of bacterial-derived products in the liver, which play significant roles in the development of NAFLD as summarized in Figure 2. RSs are an energy source for symbiotic microbiota and are fermented to release SCFAs, which in turn are beneficial for the growth of colonic cells, thus enhancing the mucosal barrier function. The regulatory effects of RSs on NAFLD mainly occur in the gut, where RSs contribute to the restoration of microbiota structure, an increase in SCFA release, and enhanced gut barrier integrity. The specific mechanisms by which RSs alleviate NAFLD by promoting overall gut health are described in the following sections.

### Intake of Resistant Starches Contributes to the Modulation of the Gut Microbiota Structure

A growing body of evidence from several animal and human studies suggests a direct causal link between NAFLD and dysbiosis of gut microbiota. It has been noticed that patients suffering from NAFLD tend to have an increased intestinal permeability along with microbiota dysbiosis (12). A significantly elevated abundance of various species of gut microbiota was identified in NAFLD patients, including *Firmicutes* (i.e., *Erysipelotrichia, Lachnospiraceae*, and *Lactobacillus*) and *Bacteroidetes* (i.e., *Prevotella* and *Parabacteroides*) (38). Patients with NAFLD have a reduced population of *Bacteroidetes* and an increased proportion of *Prevotella* and *Porphyromonas spp.* compared with healthy individuals (39). In an animal model of NAFLD, decreased abundance of *Akkermansia muciniphila* was observed (40). It was also found that *A. muciniphila* prevents fatty liver disease by regulating the expression of genes that regulate fat synthesis and inflammation in the liver (41). Moreover, a recent human study showed that the intake of RSs promotes the abundance of *A. muciniphila* (42). Notably, one of the most important findings is that the microbiota of patients with NAFLD is generally enriched in gram-negative bacteria, whereas gram-positive bacterial counts are reduced, implying a reduced abundance of butyric acid-producing bacteria (7). The collective findings suggest an association between the composition of the bacterial community, the abundance of distinct taxa, and NAFLD (24).

Findings from a fecal microbiota transplantation (FMT) NAFLD mouse model showed that the gut microbiota obtained from lean mice augmented the abundance of probiotic strains, inhibited systemic inflammation, and ultimately attenuated HFD-induced steatohepatitis (43). In contrast, germ-free obese mice receiving FMT developed low-grade inflammation and hepatic macrovascular steatosis (44). The results obtained from this study demonstrated that the gut microbiota has a significant effect on the development of NAFLD, potentially related to damage to the intestinal barrier to elicit systemic inflammation and exacerbate steatosis (45). The role of the gut microbiome structure in maintaining liver homeostasis is attributed to a dynamic interaction between the gastrointestinal tract and liver.

Patients suffering from NAFLD are affected by the structural disruption of intestinal microbes via the GLA. Hence, restoration of gut microbiota structure may be beneficial for the amelioration of NAFLD. Emerging evidence from rodent and minipig models has demonstrated that RS interventions have therapeutic efficacy in attenuating HFD-induced liver damage, thereby preventing NAFLD (46). The intake of a diet rich in RSs effectively restored the composition of the intestinal microbiome (Table 2), beneficial for gut microbial communities (47). After entering the lower gut, RSs are fermented by the intestinal microbiota to release bioactive metabolites, primarily SCFAs, which contribute to improved homeostasis of host immune metabolism (48, 49). RSs are the primary energy resources for the gut microbiota, particularly for the glycolytic bacteria in the lower gut (50). The degradation of RSs by microbiomes provides SCFAs, particularly butyrate, an energy source for colonocytes to maintain the proper structure and function of the intestinal barrier (51, 52). SCFAs can travel through the gut-brain axis, across the blood–brain barrier into the central nervous system, and affect the cellular biological mechanism of neural development, thereby resulting in various physiological processes in the liver, including gluconeogenesis, insulin sensitivity, and adenosine 5′-monophosphate activated protein kinase (AMPK) activity (53, 54). Moreover, it has been found that the SCFAs pentanoate can reduce IL-17A production in CD4^+^ T cells by inhibiting histone deacetylase activity (55). Similarly, probiotics that synthesize SCFA, particularly acetate, are involved in reducing IL-17A in hepatic type 3 innate lymphoid cells (ILC3s) (56). It was also found that dietary intake of RS and decreased colonic IL-17A stimulate intestinal immune and endocrine responses that may alter liver health (48).

**TABLE 2:** Intake of resistant starch (RS)-induced alterations of gut microbiota structure.

RS type	Model	Bacterial flora changes	References
Rice starch-oleic acid complex	HFD-induced male Sprague–Dawley rats (non-obese)	*Bacteroidetes*↓; *Firmicutes*↑; *Bifidobacterium*↑; *Lactobacillus*↓; *Coprococcus*↑; *Roseburia*↑; *Bifidobacterium*↑; and *Butyrivibrio*↑	(47)
Purple yam RS	HFD-induced male golden hamsters	*Veillonella*↑; *Lactobacillus*↑; *Coprococcus*↑; *Allobaculum*↑; *Parabacteroides*↓; and *Dorea*↓	(99)
Maize RS	HFD-induced female ob/ob mice	*Bifidobacteriales*↑ and *Prevotellaceae*↓	(86)
Buckwheat RS	HFD-induced male C57BL/6 mice	*Lactobacillus*↑; *Bifidobacterium*↑; *Enterococcus*↑; and *Escherichia coli*↓	(65)

Notably, the abundance of butyric acid-producing bacteria is suppressed in NAFLD patients (24). This suggests that RS intervention has the potential to alleviate NAFLD features by promoting the growth of butyric acid-producing bacteria. A recent finding validated that supplementation with RS5 augments the abundance of butyrate-producing bacteria (*Coprococcus*, *Roseburia*, *Bifidobacterium*, and *Butyrivibrio*) in an HFD-induced rat model (47). Moreover, RSs derived from purple yam were found to increase the abundance of *Bifidobacteria, Lactobacillus*, *Coprococcus*, and *Allobaculum* while decreasing the abundance of *Parabacteroides* and *Dorea*. Among these, the alleviated abundance of probiotics, including *Bifidobacteria* and *Lactobacillus*, has been implicated in mitigating blood hyperlipidemia in an HFD-induced hamster model (57). Finally, intervention with green banana-derived RSs promoted the release of SCFAs and helped restore the gut microbiota structure by increasing the abundance of *Lactobacillus*, *Bifidobacterium*, and *Enterococcus*, while inhibiting the growth of *Escherichia coli*, which resulted in ameliorating NAFLD in an obese mouse model (35). Despite these findings, the molecular basis underlying the observed anti-NAFLD effect of RSs mediated by maintaining gut microbiota structure and released SCFA is still not well established.

In addition to maintaining the gut microbiota structure, RS intervention also contributes to enhanced gut barrier function and regulation of BA metabolic homeostasis, as well as the reduction of harmful metabolites produced by intestinal pathogens (58, 59). RSs were found to bind to BAs with high affinity, resulting in suppressed BA reabsorption in the colon and lowered intestinal cholesterol absorption (60). Furthermore, symbiotic bacteria can exploit RS fermentation to produce bacterial metabolites that prevent colonic mucin depletion, thus maintaining healthy mucosa (25, 61). Mucin, in turn, promotes host-microbe symbiosis and enhances gut barrier integrity. Taken together, RS supplementation potentially regulates the release of various metabolites by symbiotic bacteria, including bioactive peptides, BAs, and EnEth, which are beneficial to the host as vital modulators of immunometabolism (62–66). This further indicated that dietary RSs can alleviate NAFLD through the GLA.

### Regulatory Activity of Metabolites Derived From the Gut Microbiota Fermentation of Resistant Starches in the Gut-Liver Axis Toward Alleviating Liver Damage in Non-alcoholic Fatty Liver Disease

#### Regulatory Role of Short-Chain Fatty Acids Upregulated by the Intake of Resistant Starches

The intestinal barrier protects the host against bacterial invasion while harboring commensal bacterial colonization in the lower gut. Hence, a functionally intact intestinal barrier plays a vital role in sustaining overall host health. The metabolites released by the fermentation of commensal bacteria mainly contain a variety of FFA SCFAs (i.e., acetate, propionate, and butyrate), an energy source for intestinal epithelial cells and, more importantly, key molecules involved in regulating chemo-sensing activities and subsequent cell signaling cascades in the intestinal mucosal layer, thereby sustaining gut homeostasis (67). Numerous studies have established that SCFAs are involved in regulating immune-metabolic homeostasis by activating metabolite-sensing G-protein coupled receptors (GPCRs) (67, 68). A recent study demonstrated that GPR41 and GPR43 regulate molecular events associated with inflammation, gut homeostasis, and metabolic alterations (69). Moreover, GPR43 activation has the potential to improve hepatic steatosis associated with high-fat obesity (70). Both GPR41 and GPR43 can be activated by acetate, butyrate, and propionate to regulate molecular events associated with inflammation, gut homeostasis, and metabolic alterations (69). In the liver, SCFAs stimulate GPR41 and GPR43 to activate AMPK in a peroxisome proliferator-activated receptor (PPAR)-γ-dependent manner, leading to regulation of hepatic glycolipid homeostasis via increased hepatic lipid oxidation (71). SCFA-induced serotonin release from enterochromaffin cells can influence gastrointestinal motility (72, 73). SCFAs might directly influence the brain by crossing the blood-brain barrier, reinforcing blood-brain barrier integrity, modulating neurotransmission, increasing anorexigenic neuropeptide expression, and enhancing satiety (74, 75). RS in mice markedly increases gut microbiome-derived tryptophan, a precursor of serotonin that can cross the blood–brain barrier and increase the production of cerebral serotonin; this means that the more RS intake in the diet, the more satiety can be enhanced by promoting SCFAs production while reducing caloric intake. Altogether, intake of RSs can lead to increased SCFAs by promoting mucus secretion, enhancing intestinal epithelial tight junctions (TJs), preventing dysbiosis of the intestinal microbiota, preventing endotoxins, and reducing caloric intake, inflammation, and oxidative stress in the liver, thereby lowering the risk of developing NAFLD.

#### Resistant Starches Exerting Modulatory Effects on Bile Acid Metabolism and Signaling

There is increasing evidence that a high correlation exists between BAs and SCFAs, and that their cross-talk involves the regulation of the interactive physiological status between the liver and the intestine (76). BAs may exist as primary BAs [i.e., chenodeoxycholic acid (CDA) or cholic acid (CA)] produced as glycine or taurine conjugates in the liver, and secondary BAs synthesized by the gut microbiota [i.e., deoxycholic acid (DCA) or lithocholic acid (LCA)] (76). Most gram-positive gut bacteria (i.e., *Clostridium*, *Enterococcus*, *Bifidobacterium*, and *Lactobacillus*) with bile salt hydrolase activity can produce secondary BAs (77). As previously mentioned, RS intake increases the excretion rate of primary BAs, leading to lowered blood LDL and total cholesterol levels. Meanwhile, RSs were found to contribute to the enhanced release of secondary BAs because of the increased abundance of *Lactobacillus* and *Bifidobacterium* (35). However, at high physiological concentrations, secondary BAs negatively affect the gut by augmenting oxidative stress and stimulating apoptosis and mutations, resulting in an increased risk of developing colon cancer (76, 78, 79). In contrast, a moderate level of secondary BAs inhibits colonic inflammation by downregulating pro-inflammatory cytokines (80). Reduced levels of secondary BAs and their production *Ruminococcaceae* have been detected in ulcer colitis (UC) patients, and supplementation with secondary BAs has been shown to ameliorate disease status in a TGR5 dependent manner (66, 79). It is worth noting that TGR5 activation significantly suppressed the TLR4/NF-κB pathway against inflammatory damage in the liver (81). Below toxic concentrations, a higher proportion of secondary BAs may inhibit the adipogenesis pathway and enhance bile flow in the liver, which is beneficial for preventing NAFLD (82). A recent study revealed that RSs derived from green bananas contributed to the increased abundance of *Ruminococcaceae* (83). This suggests a complex and integrated link between the gut microbiota and their metabolites, which collaboratively govern the host immune-metabolic responses along the GLA and related physiological alterations by the actions of GPCRs such as TGR5 or FXR. In this case, the RS-induced bacterial metabolites in the lower gut played a key role in regulating immune metabolism homeostasis via integrated cellular, molecular targets and mediated pathways along the GLA, eventually improving liver physiological functionality.

#### Resistant Starches Modulating the Molecular Events Involved in Immune-Metabolic Homeostasis in the Liver

The symbiotic relationship and communication between the host and gut microbiota are believed to occur via exchange of signals of bacteria-produced metabolites and molecular biomarkers synthesized by the host. The intake of RSs may have therapeutic efficacy in maintaining liver function by providing beneficial metabolites produced from colonic fermentation. More specifically, RS supplementation was shown to significantly promote the release of fecal butyrate, which has anti-inflammatory properties in the intestinal epithelium to maintain mucosal immune tolerance and enhance intestinal barrier functions by acting as a histone deacetylase (HDAC) inhibitor or signal molecule targeting GPCRs (58). It has been demonstrated that the uptake of butyrate and its synthetic derivative, N-(1-carbamoyl-2-phenyl-ethyl) butyramide (FBA), in the liver can enhance fatty acid oxidation by activating AMPK-acetyl-CoA carboxylase against fatty liver (84). In addition, butyrate exerts protective effects by activating the GPR43/β-arrestin-2/NF-κB network against LPS-induced liver injury in a mouse model (85).

Furthermore, RS intake plays a key role in maintaining BA homeostasis. A recent finding highlighted that consumption of maize RSs increased the biosynthesis of secondary BAs that enhanced cholesterol homeostasis, resulting in the mitigation of the metabolic syndrome of obesity in a dose-dependent manner (86). However, the underlying mechanism needs to be further elucidated. Unconjugated or secondary BAs can bind to a variety of receptors, including FXR, pregnane X receptor (PXR), and TGR5, to initiate signal transduction involved in regulating CYP450 enzymes, suggesting a regulatory role for BAs in host xenobiotic metabolism (87). These findings suggest that a feedback loop exists between secondary BAs and the gut microbiota structure, which is implicated in the modulation of host immune responses, energy, and xenobiotic metabolism. This ultimately results in the regulation of liver metabolic homeostasis, suggesting that dietary RSs can be a potential therapeutic strategy for NAFLD.

#### Resistant Starches Exerting Modulatory Effects on Endogenous Ethanol

Previous studies have shown that RSs have a positive effect on intestinal flora dysbiosis and a significant inhibitory effect on the EnEth content; the latter is higher in patients with NAFLD than in healthy individuals (43). The gut microbiomes from NAFLD patients are enriched in *E. coli*, which produces a high level of EnEth (88). A recent study indicated that EnEth can upregulate the expression of inflammatory cytokines and thus increase intestinal permeability to compromise intestinal barrier function (11). In addition, EnEth impedes the tricarboxylic acid cycle, which promotes fatty acid synthesis and exacerbates hepatic steatosis (5). Previous studies have shown that RSs have a significant modulatory effect in preventing dysbiosis by inhibiting the growth of intestinal pathogens such as *E. coli* (64, 65). Taken together, RSs may inhibit EnEth production by restoring the dysbiotic gut microbiome, thereby preventing hepatic steatosis. However, studies on the molecular basis and functional implications of EnEth in the GLA are limited. Therefore, it would be worthwhile to study this in the future.

### Intake of Resistant Starches Modulates Energy Homeostasis and Related Hormone Signaling

Resistant starch supplementation has been clinically proven to effectively modulate metabolic endotoxemia, insulin resistance, and oxidative stress in patients with T2DM, implying a strong therapeutic potential of RSs (89). As the intake of RSs can improve the production of SCFAs from gut bacterial fermentation, SCFAs, key colonic metabolites of RSs, act as signaling molecules to regulate appetite and maintain glucose metabolic homeostasis by upregulating proglucagon and pro-peptide YY (PYY) gene expression, increasing the levels of plasma glucagon-like peptide (GLP)-1 and PYY, two gut secreted hormones (90). GLP-1 is an anorexigenic intestinal hormone secreted by the intestinal endocrine cells that primarily controls nutrient and food intake. RS supplementation also induces the secretion of GLP-1 and PYY to inhibit body fat accumulation in mice (91). The molecular basis of the RSs triggering GLP-1 secretion depends on the interactions of SCFAs with GPR43, similar to that of the FFA receptor (FFAR)-2 through ligand binding (63). Findings from a FFAR2^–/–^ mouse study demonstrated that colonic fermentation of inulin increases the secretion of GLP-1 and PYY in an FFAR2-dependent manner (92). Moreover, a GLP-1 agonist was shown to restore insulin sensitivity and reduce hepatic TC, TG, and LDL-C levels, suggesting the anti-obesity potential of GLP-1 (10, 93). As previously mentioned, SCFAs, particularly propionate, can stimulate intestinal enteroendocrine cells to release PYY, which is involved in the modulation of electrolytes and water absorption in both epithelial and neuronal cells (94, 95). The above findings suggest that FFA receptors play a crucial role in sensing the release of SCFAs from colonic fermentation of RSs to regulate the secretion of the intestinal hormones GLP-1 and PYY. The release of GLP-1 and PYY in turn inhibits appetite and food intake to prevent obesity. This finding implies a possible regulatory effect of RSs consumption on glucose homeostasis. Nonetheless, the molecular basis underlying RS intake regulation of gut hormone secretion and subsequent metabolic outcomes is not fully understood; therefore, further investigations are warranted.

## Conclusion and Perspectives

**FIGURE 3:**
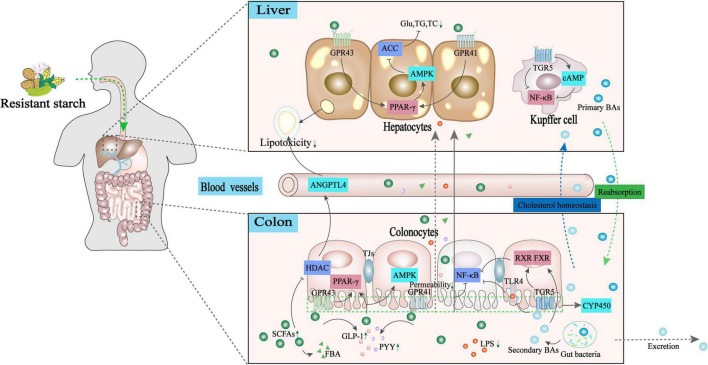
Potential mechanisms underlying RSs regulating the GLA immune-metabolic homeostasis toward NAFLD mitigation. The gut bacterial metabolites released after intake of RSs act as crucial molecules that interact with a broad range of sensing receptors along with the GLA. (1). Secondary BAs bind to TGR5/FXR receptors in the intestine, inhibiting the TLR4/NF-κB inflammatory signaling pathway to ameliorate NAFLD. (2). SCFAs binding with GPR41/GPR43 receptors in the intestine and hepatic activates PPAR-γ/AMPK signaling pathway to inhibit acetyl CoA carboxylase. As such, the production of glucose triglyceride and total cholesterol production is inhibited. (3). SCFAs can enhance the release of pro-peptide YY (PYY)/glucagon-like peptide 1 (GLP-1) by ligand binding with GPR43, which contributes to regulating appetite to maintain energy homeostasis. (4). SCFAs act as a histone deacetylase inhibitor to strengthen intestinal barrier functions, or elevate angiopoietin-like 4 secretions to reduce lipotoxicity and inflammation by potentially activating PPAR-γ. ACC, acetyl-CoA carboxylase; AMPK, adenosine 5′-monophosphate (AMP)-activated protein kinase; ANGPTL4, recombinant human angiopoietin-like protein 4; BAs, bile acids; CAT, catalase; FBA, N-(1-carbamoyl-2-phenyl-ethyl) butyramide; FXR, farnesoid X receptor; GPCR, G protein-coupled receptors; GLP-1, glucagon-like peptide 1; GPR43, G protein-coupled receptor 43; HDAC, histone deacetylase; NF-κB, nuclear factor kappa-B; PGC1α, peroxisome proliferator-activated receptor-γ coactivator-1α; PPAR-γ, peroxisome proliferator activated receptor-γ; SCFAs, short-chain fatty acids; SOD, superoxide dismutase; TC, total cholesterol; TG, triglyceride; TLR4, toll-like receptor 4; PYY, pro-peptide YY.

Non-alcoholic fatty liver disease imposes a substantial economic burden on developing or developed countries worldwide. The high prevalence of NAFLD and less effective pharmaceutical treatments have led to new and alternative therapeutic approaches for NAFLD based on multiple factors, including dietary impact, gut microbiota structure, hormone secretion, and intestinal and systemic immune responses (96). The mechanism underlying the development of impaired liver function depends on the host-microbe-metabolic interplay along the GLA, a critical basis for rationalizing the use of dietary supplementation as a therapeutic strategy. A review of recent literature shows that dietary RSs, as prebiotics, contribute to the restoration of a healthy gut microbiota structure, beneficial for maintaining gut barrier integrity and mucosal immune tolerance. This eventually leads to the prevention of pathogenic invasion and endotoxemia-mediated metabolic syndrome. Moreover, gut bacterial metabolites released after RS intake promote the growth of intestinal epithelial cells and act as key molecules that interact with a broad range of sensing receptors along the GLA, including GPR41, GPR43, FXR, PXR, and TGR5. Upon ligand binding, SCFAs and secondary BA elicit a series of signaling cascades in the intestine and liver to sustain immune metabolic homeostasis (Figure 3). These findings strengthen our understanding of how interactions between the gut microbiota and host regulate immune-metabolic crosstalk in the GLA at the molecular level. This provides insights into the dietary-immune-metabolic interplay by which the gut microbiome profiles and immune-metabolic homeostasis are well maintained. However, existing studies on the health-promoting effects of RSs on NAFLD are still scarce; thus, the differences among various types of RSs in NAFLD prevention are unclear. In addition, a variety of microbiota-derived metabolites may permeate the blood–brain barrier and enter the central nervous system; however, their implication in the pathogenesis of NAFLD is still unknown. Future research on the role of RSs in NAFLD should focus on the following: (1) elucidating the effect of different types of RSs and the roles of their distinct metabolite profiles after colonic fermentation; (2) analyzing the effect of the particular metabolic profile of different RSs on microbiota composition at the species level; and (3) understanding the relationship between specific RSs and typical gut microbial strains and how they modulate factors associated with NAFLD.

## Author Contributions

WZ: conceptualization and writing the review. YZ: data collection and original draft of the manuscript. RT: writing the review and editing. HD: writing and funding acquisition. HZ: project administration, manuscript revising and editing, and funding acquisition. All authors contributed to the article and approved the submitted version.

## Conflict of Interest

The authors declare that the research was conducted in the absence of any commercial or financial relationships that could be construed as a potential conflict of interest.

## Publisher’s Note

All claims expressed in this article are solely those of the authors and do not necessarily represent those of their affiliated organizations, or those of the publisher, the editors and the reviewers. Any product that may be evaluated in this article, or claim that may be made by its manufacturer, is not guaranteed or endorsed by the publisher.
